# Frequency Dependence of FINEMET/Ni/G Composite Ribbons Coated with As-Grown Graphene Layer via Chemical Vapor Deposition

**DOI:** 10.3390/nano15171310

**Published:** 2025-08-25

**Authors:** Yupo Wu, Yijun Chen, Zhenjie Zhao, Yenan Song

**Affiliations:** 1 Engineering Research Center for Nanophotonics and Advanced Instruments, Ministry of Education, School of Physics and Electronic Science, East China Normal University, Shanghai 200241, China; 2 Joint Institute of Advanced Science and Technology, East China Normal University, Shanghai 200062, China

**Keywords:** low-temperature CVD, as-grown graphene layer, giant magneto-impedance, FINEMET composite ribbon, magnetron sputtering

## Abstract

Enhanced Giant Magneto-Impedance (GMI) effects of composite materials play a crucial role in producing devices with a good soft magnetic property. To improve this soft magnetic property, graphene is introduced to increase the conductivity of composite materials. However, the quality of graphene layers restricts the enhancement of GMI effects. There are few reports on the direct growth of graphene on Fe_73.5_Si_13.5_B_9_Cu_1_Nb_3_ (FINEMET). In this paper, the composite ribbons of FINEMET coated with as-grown graphene are prepared by chemical vapor deposition (CVD), which is much better than previous results obtained by methods such as the transfer method or electroless plating in quality. The Ni layer, with good magnetic conductivity, is induced to the FINEMET as an auxiliary layer by the magnetron sputtering method for high-quality graphene-layer growth due to its high carbon dissolution rate. The results show that the growth temperature of the as-grown graphene layer on the FINEMET with the best GMI ratio could reach as high as 560 °C. Moreover, it was found that an Ni layer thickness of 300 nm has a crucial impact on GMI, with the maximum ratio reaching 76.8%, which is 1.9 times that of an initial bare FINEMET ribbon (39.7%). As a result, the direct growth of graphene layers on FINEMET ribbons by the CVD method is a promising way to light GMI-based devices.

## 1. Introduction

Magnetic sensors, as a new electrical device, play a great role in modern industry, such as in geomagnetic measurements, tracking systems, anti-theft systems, and magnetic labels [1,2,3,4]. Magnetic sensors based on giant magneto-impedance (GMI) have more attractive applicational value due to many advantages, such as high sensitivity, remote query, and low manufacturing costs [5,6]. When there is a conductive or magnetic material around the soft magnetic material, the properties of materials will be altered for interactions between them. Obviously, there is a strong relationship between magnetic performance of soft magnetic materials and the surrounding coating layer, which can be studied via GMI effects. Therefore, it is necessary to study the influence of conductive or magnetic materials on the sensing element of GMI sensors, and this has become a hot spot in experimental research. Theoretically, the sensing element of a qualified GMI sensor needs to have a great magnetic impedance effect. Some factors, like measurement temperature [7,8] and structural design [9,10], also have a giant impact on GMI effects. From double-layer structures, such as Fe-Ni [11,12] and Fe-Cu-Nb-Si-B [13], to multi-layer structures [14,15], progress in structural design has reduced applicational frequency and effectively improved the impedance ratio to a certain extent. However, there are still few studies on the regulation of GMI by coating new lightweight carbon materials with soft magnetic materials, especially graphene.

Graphene is a two-dimensional material composed of mono-layered carbon atoms, which is a periodic hexagonal lattice honeycomb [16,17]. Graphene has excellent optical, electrical, magnetic, and mechanical properties, so it has wide applicational prospects [18,19,20,21,22]. Recently, some researchers reported that high-quality graphene could be coated onto amorphous or nanocrystalline ribbons to develop a late-model composite, which shows promising performance on GMI effects. For example, Y. Chen et al. [23,24] systematically studied the morphology, structure, and magnetism of FINEMET/G composite ribbons and confirmed that graphene does regulate the magnetism of metal belts. Moreover, the same team developed a sandwich FINEMET/RGO/FeCo composite ribbon structure with significantly enhanced giant magneto-impedance. Y. Zhang et al. [25] found that the vertical growth morphology of graphene can greatly increase magneto-impedance effects of Fe_75.5_Cu_1_Nb_3_Si_13.5_B_7_ composite strips by changing the growth morphology of graphene on the composite metal strip. Z. Yang et al. [26] studied micro-patterned Co-based amorphous ribbons coated with single-layer graphene and found that their giant magneto-impedance effect was enhanced by 13.1%. It is explained that this is the result of reducing the strip surface roughness and the closed magnetic flux path below the graphene film layer. These studies all strongly confirm the regulatory effect of graphene layers on GMI effects.

Experimental studies further prove that the quality of graphene is a particularly important factor for the regulation of GMI. So far, some constructive routes of graphene production have been established, such as micro-mechanical exfoliation [27], chemical vapor deposition (CVD) [28,29,30], the reduction of graphene oxide (RGO) solutions [31,32] etc. The quality of graphene is strongly related to purity, defects, and the number of graphene layers. Thus, the quality of RGO produced by a redox reaction is not optimal due to excessive impurities that are introduced. In addition, incomplete reduction also causes residual oxygen-containing groups, defects, and folds. On the contrary, the CVD method can effectively reduce the introduction of impurities so that the quality of graphene is higher than that of the former and can be comparable to the graphene prepared by the stripping method. The method of growing graphene directly on FINEMET bands by CVD is worthy of an in-depth study for its innovations, and there are few related research studies to date.

In this work, growing graphene layers directly on FINEMET ribbons by CVD was achieved. By using PMMA as a carbon precursor and introducing Ni as an auxiliary transition layer for graphene growth, FINEMET/G and FINEMET/Ni/G composite ribbons with significantly enhanced GMI effects were prepared. GMI measurements and a Raman spectrometer were used to probe the mechanism of the enhanced GMI effect on FINEMET/G and FINEMET/Ni/G by a direct-growth graphene layer, while the relationship among Ni layer thickness, growth temperature, and GMI effect was also systematically analyzed and studied in detail.

## 2. Experiments

### 2.1. Preparation of FINEMET Ribbons

**Figure 1:**
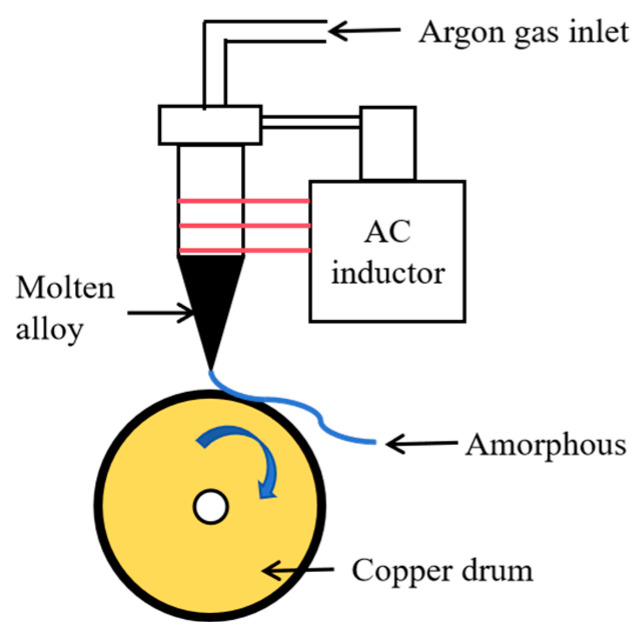
Schematic diagram of production of amorphous Fe_73.5_Cu_1_Nb_3_Si_13_B_9.5_ ribbons.

Fe-based amorphous ribbons were first prepared by the rapid quenching technique and then were cut to a size of 20 mm in length, 0.6 mm in width, and 33 µm in thickness. Next, the ribbons were annealed for 20 min to become FINEMET ribbons at 9.8 × 10^−5^ Pa and 540 °C. The preparation method is shown in Figure 1.

### 2.2. Preparation of FINEMET/G

A CVD system with a horizontal tube furnace was used to synthesize thin graphene films. The FINEMET ribbons prepared in advance were placed in a quartz boat with a non-contact roller surface (surface F) faced upward, and the whole quartz boat was placed in an area with constant temperature growth in the middle of a tube furnace. Another quartz boat containing 2 g of PMMA was placed in an area of the inlet of the tubular with a temperature of 140 °C. A vacuum background of 3 Pa was reached in the tube before carbon deposition. Then, the substrate was heated to 500 °C, 520 °C, 540 °C, 560 °C, 580 °C, and 600 °C and held for 60 min under 100 sccm N_2_/H_2_ (5 atm% H_2_). The temperature for growing graphene layers was held for 60 min and then cooled down to room temperature.

### 2.3. Preparation of FINEMET/Ni/G

The preparation process of the FINEMET/Ni/G composite ribbons consisted of magnetron sputtering of Ni layers and LPCVD growth of graphene layers. The vacuum chamber was pumped to a pressure below 5.0$\;\times 10^{-4}$ Pa before sputtering. The Ni layers with different thicknesses were sputtered onto FINEMET ribbons under 25.5 sccm Ar at 60 W and 1.9 Pa. By adjusting the time for sputtering, FINEMET/Ni composite ribbons with a nickel-plating time of 10 min, 30 min, 50 min, 70 min, and 90 min were prepared. The thickness of the Ni layers was measured by a step profiler (DEKTAK XT, Bruker, Ettlingen, Germany). The process for LPCVD growth was similar to that used for preparing FINEMET/G, but the CVD growth temperature of all composite ribbons was set to 560 °C.

### 2.4. Characterization

**Figure 2:**
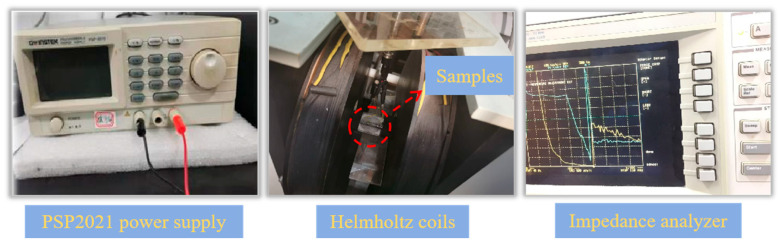
The GMI effect test system used in this experiment.

Images of the samples obtained by a field emission scanning electron microscope (FESEM) were taken with a Gemini450, Zeiss, Jena, Germany The quality of graphene was determined by a Raman spectrometer (HR Evolution, Horiba, Kyoto, Japan) with a laser wavelength of 532 nm. GMI measurements were carried out using a precision impedance analyzer (HP4294A, Agilent, Santa Clara, CA, USA). The rms value of the ac driving current was kept constant at 20 mA, and its frequency ranged from 100 Hz to 100 MHz. During the test, the longitudinal axis of the sample was parallel to the external magnetic field. The relative change of the MI ratio was defined as follows:

(1) $$\frac{∆Z}{Z}=\frac{Z\left(H_{ex}\right)-Z\left(H_{max}\right)}{Z\left(H_{max}\right)}\times 100\%$$

where Z(H_ex_) and Z(H_max_) were the impedance values of the samples in the external field and the maximum magnetic field, and Z(H_max_)is 90 Oe. The instrument shown in Figure 2 is the giant magneto-impedance effect testing system used in this experiment.

## 3. Results and Discussion

### 3.1. Micro-Structure, Morphologies, and GMI Measurement of FINEMET/G

**Figure 3:**
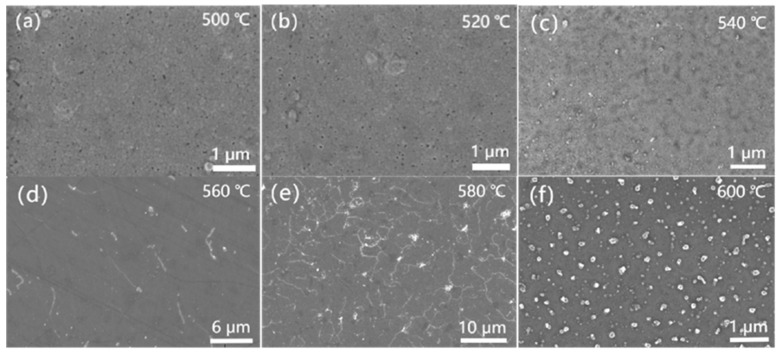
SEM images of FINEMET/G composite ribbons at (**a**) 500 °C, (**b**) 520 °C, (**c**) 540 °C, (**d**) 560 °C, (**e**) 580 °C, and (**f**) 600 °C.

Figure 3a–f show the SEM images of the different FINEMET/G ribbons synthesized at various temperatures. Figure 3a–f show images obtained at six different temperatures, from 500 °C to 600 °C. The clear contrast difference shown in Figure 3a–f werethe distribution area of Fe (Si) crystallite. The coverage area of crystallite became wider, which accounted for a change in the micro-structure of the FINEMET below the graphene layers as the temperature increased. It can be seen that the degree of crystallization is very low in Figure 3a–c, which show a flat and smooth surface morphology of the FINEMET/G ribbons. When the temperature reached 560 °C, the surface morphology of the FINEMET/G became flatter and smoother and had better uniformity, which is shown in Figure 3d. The main reason for the surface morphology of the different ribbons behaving differently was the crystallization coefficient and degree of crystallization, which depend on temperature. According to a previous study [33], the crystallization coefficient of Fe-based amorphous ribbons annealed at 540 °C for 30 min is 82%, while the crystallization coefficient at 650 °C may be up to 100%. These findings suggest that a higher temperature leads to a larger crystallization coefficient in the FINEMET; thus, excessive crystallization led to magnetic exchange decoupling in the samples and caused the loss of good soft magnetic properties. From Figure 3e, obvious crystallinity can be seen at 580 °C, indicating that more $\alpha -Fe\left(Si\right)$ crystallites were formed. A similar phenomenon is also seen in Figure 3f when the temperature reaches 600 °C. In conclusion, the flat surface of the FINEMET provided an extremely good environment for the growth of high-quality graphene layers, and it was obvious that the best condition for the growth of high-quality graphene layers by CVD was 560 °C.

**Figure 4:**
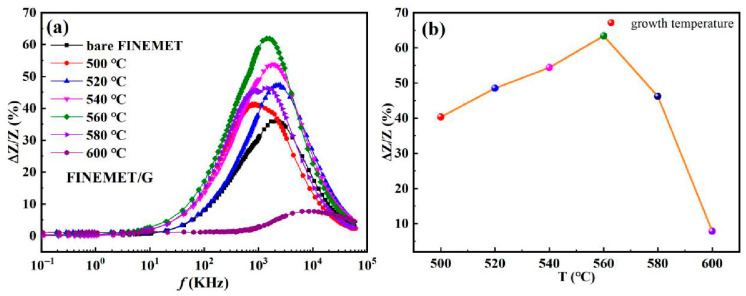
The frequency dependence of GMI ratio (**a**) of FINEMET/G composite ribbons prepared at different CVD growth temperatures and (**b**) their maximum impedance ratios.

Figure 4a depicts the impedance ratio of the FINEMET/G prepared under different temperature conditions. At all temperatures, the impedance ratio of the FINEMET/G ribbons increased rapidly with the increase in frequency in the beginning; then climbed to the peak of the curve, reaching the maximum at *f_max_*; and finally decreased as the frequency increased. All curves with different marks showed a similar change, which reflected on the relationship between the impedance ratio and frequency. The characteristic frequency (*f_max_*) was a significant frequency that allowed the GMI ratio to reach the maximum value. The characteristic frequency can be roughly estimated as follows [34]:

(2) $$f_{max}=f\left(\frac{1}{u_{T}\pi d^{2}\sigma }\right)$$

where *μT*, *d*, and *σ* are the dynamic transverse permeability, thickness, and conductivity of the composite ribbons, respectively. In the case of weak skin effects, the external field was insufficient to induce a significant change in impedance.

As the frequency increased, both the domain walls’ motion and magnetization rotation contributed to the dynamic transverse permeability, which boosted the GMI response. However, according to the electromagnetic model of GMI effects [35,36], when the frequency increases to tens to hundreds of MHz, which is bigger than the *f_max_*, the domain wall motion is restrained by eddy current damping with an increase in eddy current loss; thus, effective transverse permeability decreases. When the driving frequency continues to rise at a higher frequency, the damping of the eddy current enhances, and the magnetization caused by the domain wall motion gradually dies down; thus, the magneto-impedance ratio is weakened due to a decrease in transverse permeability.

Figure 4b depicts the relationship between the maximum GMI ratio at the characteristic frequency and growth temperature. It is notable from the line chart in Figure 4b that the maximum GMI ratio initially increased with the growth temperature until 560 °C and decreased in the end. Obviously, the maximum impedance ratio reached the maximum (61.9%) at 560 °C. In the range of 500 °C to 560 °C, the graphene layer could achieve better quality in the skin layer. As the temperature increased, the surface morphology of the FINEMET/G became flatter and smoother and had better uniformity. As mentioned earlier, the FINEMET/G had the best crystalline state at 560 °C, where the soft magnetic properties could be kept in extremely good condition. In this case, the conductivity and electron mobility of the composite ribbon became better, forcing the current to concentrate on the surface, which made the resistance of the skin layer come down. As a result, the current was more concentrated on the FINEMET/G surface so that the enhanced skin effect led to the enhancement of GMI. However, the maximum impedance ratio fell quickly after 560 °C. As mentioned above, the crystallization coefficient, related closely to the micro-structure, was greatly affected by the growth temperature. When the ambient temperature was higher than 560 °C, the Fe-B phase under the graphene layer went through a relaxation and crystallization process, resulting in a decrease in the maximum impedance ratio, and it made the soft magnetic properties worse as the temperature increased. In conclusion, the results shown in Figure 4a,b indicate that 560 °C was the optimal value for the best GMI effect, which is the same as what we see in Figure 3.

### 3.2. Microstructure, Morphologies, and GMI Measurement of FINEMET/Ni/G

**Figure 5:**
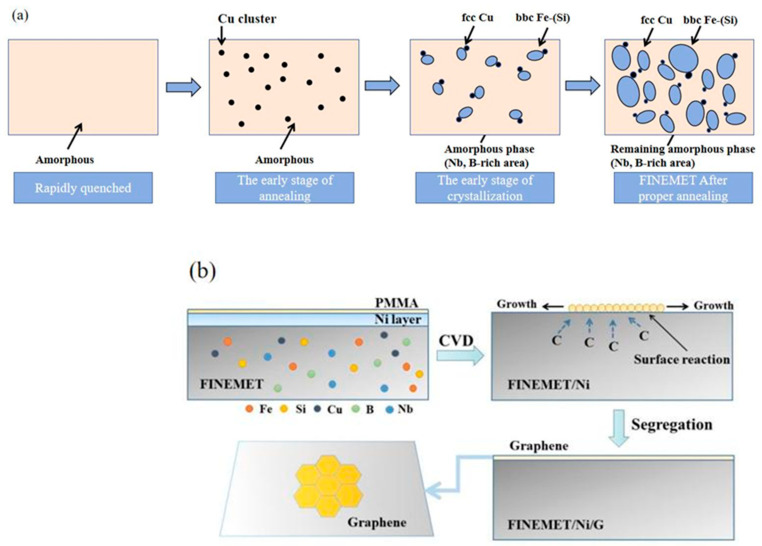
(**a**) Preparation process of FINEMET and (**b**) schematic diagram of as-grown graphene layer on FINEMET/Ni.

In order to improve the quality of graphene layers, Ni was selected as an auxiliary transition layer for the growth of graphene layers. This is mainly due to the extremely high carbon dissolution rate of nickel. Figure 5 depicts the preparation of FINEMET/Ni/G and a schematic diagram of the as-grown graphene layer on the FINEMET/Ni by LPCVD. Firstly, the Ni layer was plated to the FINEMET by magnetron sputtering and was annealed at a suitable temperature to heighten the crystallinity of the FINEMET/Ni. In this process, Ni atoms were not integrated into the FINEMET at 560 °C but formed a flatter and denser film on the surface of the FINEMET after annealing. In this experiment, PMMA was chosen as the carbon source, and thus, the reaction needed to be carried out at a low temperature, ensuring a high decomposition rate of the carbon source. PMMA was driven by airflow at the nozzle and deposited on the substrate. At 560 °C, the PMMA was decomposed into carbon intermediate species, CH_x_ (x = 0–3), due to a reaction catalyzed by the Ni layer. Because of the high carbon solubility of Ni, a cluster of carbon atoms diffused into the bulk of the FINEMET/Ni, as was segregated on the surface later to make a surface reaction take place. Then, under the simultaneous supply of the carbon source, the graphene layer was formed directly on the surface of the FINEMET/Ni via carbon segregation, according to previous research studies on graphene growth on Ni flakes [37,38]. Finally, the FINEMET/Ni/G was prepared successfully in this way, which proved the feasibility of the direct growth of graphene layers on FINEMET. The content of the Ni atoms affected the growth quality of the graphene, so we designed a feasible experimental scheme to study the direct relationship among the thickness of Ni layers, the quality of graphene layers, and GMI.

**Figure 6:**
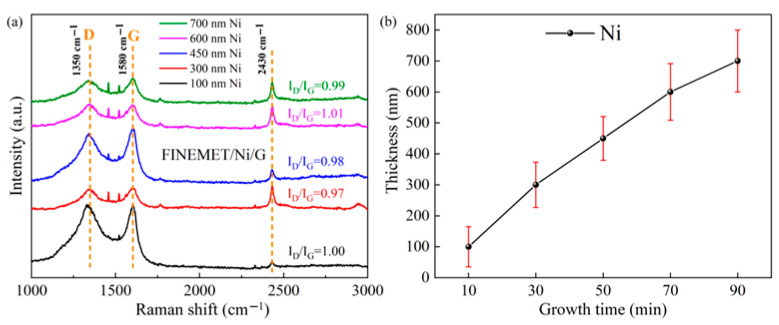
(**a**) Raman spectra of FINEMET/Ni/G composite ribbons (**b**) with different Ni thickness.

By adjusting the magnetron sputtering time, we designed some specimens with different Ni layer thicknesses. The thickness of Ni layers was measured by a step profiler, and the quality and layer number of the graphene were evaluated by Raman spectroscopy [39,40]. Figure 6a shows the Raman spectrum of the FINEMET/Ni/G composite ribbons with different Ni layer thicknesses. The relationship between the Ni layer thickness and growth time is exhibited in Figure 6b. The appearance of a graphene characteristic peak indicates that the graphene layer was synthesized successfully. The D and G characteristic peak of the graphene layer was at 1350 cm^−1^ and 1580 cm^−1^, and that of the FINEMET ribbon was at 2430 cm^−1^. There were in total five different color curves corresponding to different thicknesses of Ni layers. As shown in Figure 6a, the intensity ratios of the D and G peak (I_D_/I_G_) were generally high, indicating that the quality of the graphene was not high. This is because the higher the I_D_/I_G_ was, the more defects the graphene had. Hence, the I_D_/I_G_ of the FINEMET/Ni/G (corresponding to the red curve in this figure) was found to have the lowest value of 0.97 when the thickness of the Ni layer was 300 nm and the temperature was set to 560 °C. Under these conditions, the quality of the graphene layer was optimum, and with this comes the largest impedance ratio. This is mainly because functional group residues and structural defects cannot be completely removed on the graphene layer, resulting in a high I_D_/I_G_ ratio of graphene [41,42].

**Figure 7:**
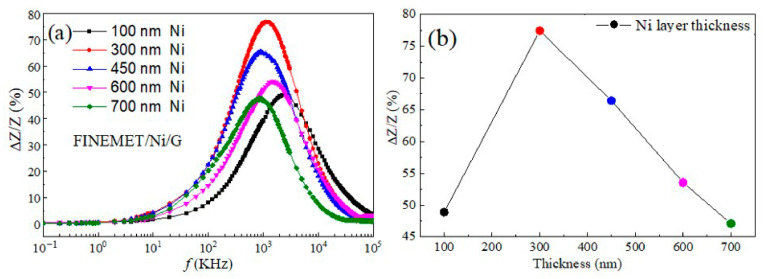
The frequency dependence of GMI ratio (**a**) of FINEMET /Ni/G composite ribbons prepared at different CVD growth temperatures and (**b**) their maximum impedance ratios.

**Figure 8:**
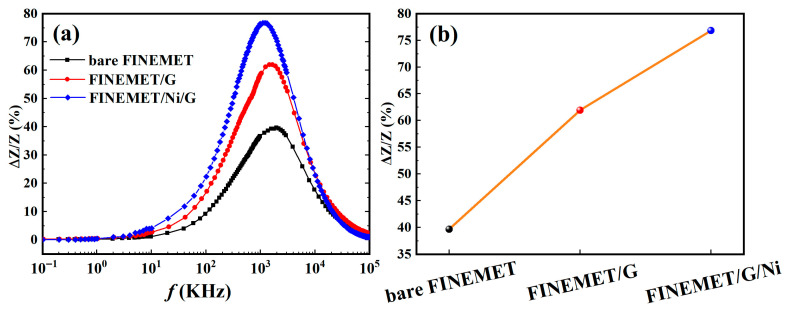
The frequency dependence of GMI ratio (**a**) and maximum impedance ratios (**b**) of bare FINEMET and FINEMET/G grown at 560 °C and FINEMET/Ni/G grown at 560 °C with a Ni thickness of 300 nm.

Figure 7a shows a chart of the GMI radio frequency dependence, which indicates the relationship between the thickness of the Ni layer and the GMI ratio. When the frequency came to the certain characteristic frequency (*f_max_*), each of the GMI ratios of the composite ribbons reached a corresponding maximum. Figure 7b depicts a line chart which shows the maximum impedance ratio with different thicknesses of the Ni layer. The average thickness of the Ni layer corresponding to different plating times is shown in Figure 7b. Apparently, the Ni plating thickness and maximum impedance ratio were not linear according to the variation trend of the broken line. The maximum GMI value of 76.8% was obtained for the FINEMET/Ni/G composite ribbons on an Ni plating thickness of 300 nm, which indicated that the soft magnetic performance of composite ribbons was optimal (Figure 8). However, if the thickness of the Ni layer continued to increase, the impedance ratio would gradually decrease. This was closely related to impedance (*Z*) and the half thickness of the FINEMET/Ni/G. According to the classical skin effect, impedance (Z) in thin magnetic ribbons can be calculated by [6]

(3) $$\;\;\;\;\;\;\;\;\;Z=R_{dc}\cdot jkd\cdot coth\left(jkd\right)$$

(4) $$\;\;\;\;\;\;\;\;k=\frac{1+j}{\delta_{m}}$$

where *R_dc_* is the dc electrical resistance, *j* is the imaginary unit, 2*d* is the thickness, and *δ_m_* is the skin depth. The main physical mechanism of the GMI effect is the skin effect. The skin depth can be calculated by

(5) $$\;\;\;\;\;\;\;\;\delta_{m}=\sqrt{\frac{2}{\mu_{T}\omega \sigma }}$$

where *σ* is the electrical conductivity, *ω* is the angular frequency of the ac current, and $\mu_{T}$ is the dynamic transverse permeability. These equations suggest a relationship between impedance (Z) and skin depth. In addition, in cases where the band width was determined, impedance (Z) could be enhanced by adjusting the skin depth to a certain value with a proper thickness of the Ni layer. Nevertheless, an excessive increase in the thickness of the composite ribbons caused an increase in the eigenvalues determined by the thickness. If the thickness was increased to the width of the film which was less than the critical value, the magnetic leakage passing through the conducting layer would cause a marked decline in the impedance ratio.

According to the previous data analysis, the GMI performance of FINEMET/Ni/G with Ni layers was better than that of FINEMET/G without Ni layers, which could be explained by the following two aspects: Firstly, Ni, as a transition layer, improved the unevenness of the surface of the FINEMET ribbons, making the substrate flatter, which was helpful for improving the quality of the graphene layer synthesized by CVD. High-quality graphene has better conductivity, so it amplified the function of graphene as a conductive layer and increased the surface current density of the FINEMET/Ni/G, which caused a greater skin effect. Furthermore, the Ni layer was a material with good magnetic permeability, so the dynamic transverse permeability of the ribbons increased, and the skin depth decreased with the addition of the Ni layer, which led to an increase in impedance (Z).

**Figure 9:**
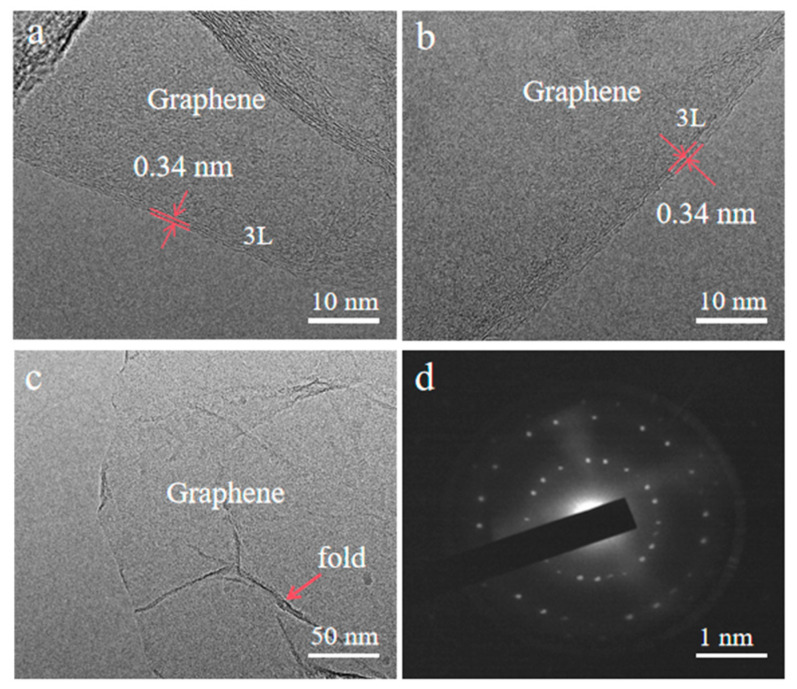
(**a**–**c**) TEM images and (**d**) SAED image of as-grown graphene film on FINEMET/Ni/G grown at 560 °C with a Ni thickness of 300 nm.

To sum up, it was noted that the best GMI effect, which reached 76.8%, could be obtained when the thickness was around 300 nm. It is clear that the FINEMET/Ni/G in this condition had a better reaction in scaling up the GMI ratio compared with the bare FINEMET (39.7%) and FINEMET/G (61.9%) at the same temperature (560 °C), which is shown in Figure 9.

To investigate the quality of as-grown carbon films on FINEMET/Ni/G, the TEM was used to test the layer numbers of the graphene. As shown in Figure 9a–c, the thickness of graphene film was from 1–3 nm, which represents a layer number from 2–6. The specific number of layers of graphene can be seen from the SAED diagram in Figure 9d. Furthermore, the crystalline of the graphene was also obtained as layered configurations.

## 4. Conclusions

In this paper, FINEMET/Ni/G composite ribbons were prepared by magnetron sputtering and LPCVD. The effects of growth temperature and thickness of the Ni layer on the appearance, structure, GMI, and magnetic properties of the composite ribbons were analyzed by a step profiler, SEM, Raman spectra, and an impedance analyzer in detail. As a result, when the growth temperature was 560 °C and the Ni layer thickness was 300 nm, the corresponding composite ribbon had the largest GMI ratio (76.8%), which was 1.9 times that of the initial bare FINEMET ribbon (39.7%). The results indicate that the soft magnetic properties of nanocrystalline ribbons could be maintained by LPCVD growth at 560 °C, which made the substrate more suitable for attaining a larger crystal. Moreover, Ni, as a transition layer, improved the unevenness of the surface of the FINEMET ribbon, which was helpful for improving the quality of the graphene layer synthesized by CVD. The high-quality graphene layer had better conductivity and increased the surface current density of the FINEMET/Ni/G, which led to a greater skin effect. These research results offer a promising approach for the design and preparation of sensitive components based on GMI effects, which might be applied in the field of magnetic sensor applications.

## Data Availability

The original contributions presented in this study are included in the article. Further inquiries can be directed to the corresponding author.
